# Effect of cranberry bean powder and protein isolates with different drying methods on rheological properties of chicken breast myofibrillar protein gels

**DOI:** 10.5713/ab.250747

**Published:** 2026-03-11

**Authors:** Muhammad Rifqi Ananta, Koo Bok Chin

**Affiliations:** 1 Department of Animal Science, Chonnam National University, Gwangju, Korea

**Keywords:** Cranberry Bean, Drying Methods, Myofibrillar Protein, Protein Isolate, Protein Structure, Rheological Properties

## Abstract

**Objective:**

This study aimed to assess the interaction between chicken myofibrillar protein and cranberry bean powder (CBP) processed through different drying methods, including oven-drying (OD) and freeze-drying (FD), along with cranberry bean protein isolates (CBPI), on the rheological properties of chicken myofibrillar protein gels (MPGs).

**Methods:**

Two experiments were done in this study. The isolated MPG treated with various levels of CBP in different drying methods was prepared for the first experiment. The MPG with CBP and CBPI with different drying methods was prepared and compared to soy protein isolate (SPI) in the second experiment. The cooking yield (CY), gel strength (GS), viscosity, sodium dodecyl sulfate-polyacrylamide gel electrophoresis (SDS-PAGE), and microstructure were measured in both experiments.

**Results:**

The results showed that freeze-drying isolate (FDI) exhibited the best GS, CY, and viscosity, all of which were comparable to those of SPI. FDI demonstrated superior protein-protein interactions, as evidenced by SDS-PAGE and scanning electron microscopy analyses of well-formed structures. FD performed better than OD in terms of GS and viscosity but still lower than FDI. OD samples, which contained higher levels of carbohydrates and starch, displayed lower GS and less stable gel networks, indicating that starch may interfere with protein matrix formation.

**Conclusion:**

The addition of FDI or SPI was shown to be the most effective in enhancing the rheological properties of MPGs, and the higher levels of powder addition also improved viscosity, GS, and CY.

## INTRODUCTION

Chicken meat has witnessed a surge in popularity worldwide. Renowned for being a lean protein source, chicken meat offers several advantages over red meat, including lower fat and cholesterol content, ease of portioning, and fewer religious restrictions [1]. The growing demand for health-conscious options has spurred extensive research in the meat industry, leading to the inclusion of health-promoting ingredients [1]. Myofibrillar proteins (MP) from chicken breasts play a crucial role in gelation, a key factor determining the textural quality of processed meat products [2]. Myofibrillar protein gels (MPGs) play a crucial role in the texture and quality of processed meat products. These gels, primarily composed of myosin and actin, are responsible for the characteristic binding, water-holding capacity (WHC), and textural properties of many meat-based foods [3]. In recent years, there has been growing interest in incorporating plant-based proteins into meat products to enhance nutritional value, improve functionality, and address sustainability concerns [3].

The growing demand for plant-based protein additives in meat products has driven research into alternative protein sources with functional benefits. Cranberry beans (*Phaseolus vulgaris*) are nutritious legumes lauded for their high protein content, dietary fiber, and micronutrient profile. Their versatility and potential health benefits have garnered attention in various culinary and nutritional applications. One emerging application is the integration of cranberry bean powder (CBP) into food products, particularly in the development of healthier meat alternatives [4].

Different plant-based proteins exhibit unique characteristics in solubility, gelation, and water absorption stability due to their diverse structures and compositions [4]. Even when derived from the same source, variations in manufacturing or drying methods can result in powders with distinct functional attributes [4]. Drying, although beneficial for enhancing storage stability, can also induce partial protein denaturation, resulting in the formation of insoluble aggregates and altering the functional properties [5]. Protein isolates are concentrated protein fractions obtained through extraction processes, with their functional properties influenced by both the source of the protein and the processing methods used [5]. Many studies have evaluated MPGs in meat products, focusing on the interaction between MP and protein or plant-based additives. Research findings suggest that MPGs and meat products exhibit positive interactions, enhancing the overall functionality and quality of the final product [6].

Through a comprehensive analysis, this research investigates the effect of CBP and its protein isolate (CBPI) on the rheological properties of chicken MPGs. Two drying methods oven-drying (OD) and freeze-drying (FD) were applied to CBP and CBPI, respectively, resulting in four variations: oven-dried CBP (OD), freeze-dried CBP (FD), oven-dried CBPI (ODI), and freeze-dried CBPI (FDI). By evaluating these processing conditions, this study aims to investigate the interaction between chicken MPGs and cranberry bean proteins, thereby contributing to the development of functional and sustainable meat products.

## MATERIALS AND METHODS

### Preparation of cranberry bean powder

Cranberry beans imported from the USA were purchased from Dae Gu Agricultural Products. The beans were soaked in water 1:2 (w/v) to facilitate their skin removal. Peeled cranberry beans were subjected to two drying methods: OD at 60°C for 3 days and FD at −50°C for 5 days. The dried beans were ground and passed through a 100-mesh sieve (Daehan Science) to produce fine CBP.

### Preparation of chicken myofibrillar protein gels with different concentrations of oven-drying and freeze-drying

Chicken breast was purchased commercially and stored in a −70°C freezer until use. For the extraction of MP, chicken breast (200 g) was homogenized with 0.1 M NaCl in 50 mM phosphate buffer (pH 6.25) and centrifuged (2,930×g, 15 min) using a centrifuge (Supra 22K; Hanil Science Medical). After three repetitions, the obtained pellet was mixed with 0.1 M NaCl buffer (pH 6.25) and filtered to remove connective tissues using a sterilized gauze. After centrifugation (2,930×g, 15 min), the finally obtained MP pellets were collected. The protein concentration of MP was analyzed and adjusted to 2 g.kg^−1^ protein using a phosphate buffer solution [7]. Table 1 shows the formulation of chicken MPGs added with CBP (OD or FD) with different concentrations.

### Preparation of cranberry bean protein isolate

CBPI was prepared through a multi-step process following methods described by L’hocine et al [8] with slight modifications. Initially, the defatting of CBP was performed using hexane 1:2 (w/v). The mixture was stirred for 30 min, followed by centrifugation at 2,980×g for 10 min to separate the hexane from the defatted powder. The resulting defatted powder was air-dried at room temperature under a fume hood for 6 h and then oven-dried at 37°C overnight to ensure complete evaporation. For protein isolation, the defatted powder was mixed with water at a 1:15 (w/v) ratio (1 part powder to 15 parts water), and the pH was adjusted to 9.00. The mixture was then heated at 55°C for 30 min to facilitate protein extraction. After centrifugation at 10,000×g for 15 min at 4°C, the supernatant was collected, and its pH was adjusted to 4.50 to precipitate the proteins. The mixture was then centrifuged again at 10,000×g for 15 min at 4°C to separate the protein precipitate, which was filtered. The protein pellet was washed twice with a minimal amount of deionized water and centrifuged each time at 2,830×g for 10 min. The washed precipitate was resuspended in a minimum amount of water, and the pH was adjusted to 7.00. The final protein isolate was freeze-dried to obtain CBPI, which was stored at −70°C for long-term storage and further analysis.

### Preparation of chicken myofibrillar protein gels with cranberry bean powder or cranberry bean protein isolate with different drying methods

The extraction of MP and the analysis of its protein concentration were performed using the same methods as described above (see “Preparation of chicken MPGs with different concentrations of OD and FD”). Table 2 shows the formulation of chicken MPGs added with CBP, CBPI, and soy protein isolate (SPI) (as positive control), respectively.

### Proximate analysis of cranberry bean protein isolate

The moisture, protein, and lipid contents of CBP were analyzed following the methods outlined by the AOAC International [9]. The total moisture content (g.kg^−1^) was determined using the dry oven method, where each homogenized sample was placed in a filter paper thimble and dried in an oven at 100°C for 16–18 h. The crude fat content (g.kg^−1^) was assessed using the Soxhlet extraction method, utilizing the same sample prepared for moisture analysis. The sample was extracted with petroleum ether in a Soxhlet apparatus at 60°C–80°C for 5 h and then oven-dried. The fat content was then calculated based on the weight loss after extraction and drying. Crude protein content (g.kg^−1^) was determined using the Biuret method, which quantifies protein concentration based on its reaction with a copper sulfate solution to form a violet-colored complex measurable at a 532 nm wavelength.

### Myofibrillar protein gels cooking yield

MPGs were heated (20°C–80°C) in a water bath (WB-22; Daihan Scientific) for about 30 min and then cooled for 2 h in a refrigerator at 4°C. After cooling, the samples were stored at room temperature for 30 min to remove moisture. The cooking yield (CY, %) was calculated using the following formula:


(1) $$\text{CY}(\%)=\frac{\text{Sample weight after heating }(\text{g})}{\text{Sample weght before heating }(\text{g})}\times 100$$

### Myofibrillar protein gels strength

Gel strength (GS, gf) of cooked chicken MPG samples was measured using a universal testing machine (Model 3344; Instron) and Merlin software (Instron) to determine the effect of the addition of CBP and CBPI. The first peak values of the breaking force (gf) were measured by the compression test using a 500 N load cell at 50 mm/min cross-speed (9 mm diameter cylindrical probe).

### Myofibrillar protein gels viscosity

The viscosity of MPGs was determined before heating. The viscosity was expressed by recording the changes of shear stress (Pa) with increasing shear rate (1/s) in the range of 0 to 600/s using a cylinder-type rotational rheometer (RC30; Rheotec Messtechnik). The viscosity results were analyzed using Microsoft Excel (ver. 2016; Microsoft).

### Myofibrillar protein gels sodium dodecyl sulfate-polyacrylamide gel electrophoresis

Sodium dodecyl sulfate-polyacrylamide gel electrophoresis (SDS-PAGE) was performed to investigate the effect of the addition of CBP and CBPI at 5 or 10 g kg^−1^ on the polymerization of MPGs. Samples were adjusted to a 10 mg mL^−1^ protein concentration and loaded on a 10% acrylamide separating gel (0.375 M Tris, pH 8.80) and 4% stacking gel (0.125 M Tris, pH 6.80) with Laemmli sample buffer (Cat. Number 161-0737; Bio-Rad Laboratories). Electrophoresis of the samples to separate protein bands was run at 150 V for 1.5 h. A standard marker (Cat. Number 161-0318; Bio-Rad Laboratories) was used to determine the molecular weight of unknown proteins in samples.

### Myofibrillar protein gels microstructure

Microstructure was analyzed using a low-vacuum scanning electron microscope (JSM-6610LV; JEOL) to determine the three-dimensional structure of samples. Samples were diced into cubes (3 mm^3^) and fixed by soaking in 25 mg mL^−1^ glutaraldehyde in 0.1 M sodium phosphate buffer (pH 7.00) for 24 h at 4°C. The post-fixed samples were immersed in osmium tetroxide (OsO4) in a 0.1 M sodium phosphate buffer (pH 7.00) for 5 h. Afterward, samples were washed with ethanol and acetone solutions for 10 min each step of dehydration. Dehydrated samples were gold-coated using an auto sputter coater (Cressington Scientific Instruments). The microstructure of samples was observed at ×500, ×1,000, and ×2,000 magnification.

### Statistical analysis

All experiments for this study were conducted in triplicates. Data were presented as means and standard deviations using IBM SPSS Statistics software (ver. 29.0; IBM). One-way analysis of variance was conducted to analyze the difference between treatments as a factor at a significance level of 95% using Duncan’s multiple range test.

## RESULTS AND DISCUSSION

### Evaluation of cranberry bean protein isolate

#### Proximate analysis of cranberry bean powder and cranberry bean protein isolate

The proximate composition and functional properties of CBP and CBPI are influenced by processing and drying methods, as demonstrated in this study (Table 3). FD and FDI generally result in lower moisture content compared to OD and ODI, which is attributed to the efficiency of sublimation in removing water. Protein isolates (ODI and FDI) also exhibit reduced lipid content due to the defatting process, which removes non-protein components, making them more suitable for applications requiring higher protein purity. The protein content of both CBPI samples in this study was comparable to that of other legume protein isolates, which generally exceed 70% [10]. Among these, FDI shows the highest protein concentration, comparable to SPI, highlighting its potential as a plant-based protein ingredient for food formulations. These findings are consistent with research by Brishti et al [5] who reported similar effects of drying methods on the compositional properties of mung bean protein isolates. Despite comparable moisture contents, CBPI samples exhibited differences in protein content, suggesting that drying-induced changes affecting protein recovery and extractability, rather than moisture level, contributed to the observed variation. Additionally, variations in ash content reflect mineral composition differences due to processing. Overall, FD combined with protein isolation is the most effective method for enhancing protein concentration and minimizing undesirable components, making FDI a promising alternative protein source. This is supported by Boye et al [11] who demonstrate freeze-dried legume protein isolates retain superior functional properties, such as solubility, gelation, and emulsifying ability, compared to those processed using other drying methods. This highlights the benefits of FD for maintaining protein quality and functionality.

These results underscore that cranberry bean-derived ingredients, particularly FDI, offer a sustainable alternative to traditional animal-derived proteins while maintaining comparable functionality and nutritional quality. The improved protein content and functional properties of FDI could potentially enhance gelation, WHC, and emulsification when incorporated into MP systems. Further exploration into their rheological properties and interactions with MPs could lead to innovative texture modifications in meat products and the development of hybrid products with reduced environmental impact. This aligns with the growing trend of plant-based protein alternatives in the food industry, as discussed by day [12] and opens new avenues for research into the specific interactions between plant proteins and MPs in food systems. Such investigations could contribute to the development of healthier, more sustainable meat products that meet evolving consumer demands.

#### Cooking yield and gel strength

**Figure 1:**
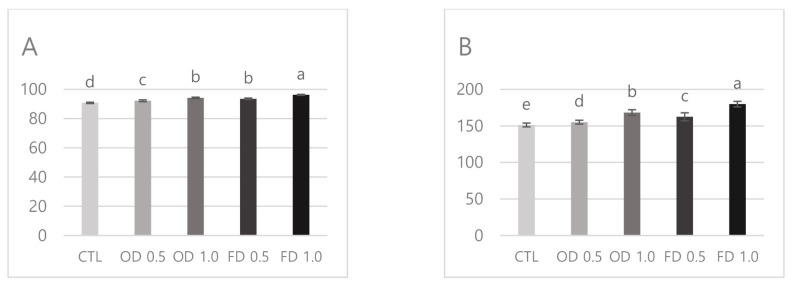
Cooking yield (%) and gel strength (gf) of chicken breast myofibrillar protein gels (MPGs) containing different levels of cranberry bean powder processed by different drying methods. (A) Cooking yield, (B) gel strength. Treatment: CTL, control; OD 0.5, MPGs added by 0.5% cranberry bean oven dried powder; OD 1.0, MPGs added by 1.0% cranberry bean oven dried powder; FD 0.5, MPGs added by 0.5% cranberry bean freeze dried powder; FD 1.0, MPGs added by 1.0% cranberry bean freeze dried powder. ^a–e^ Mean with different superscripts in the treatment are different (p<0.05).

The CY and GS of MPGs were affected by both the levels and drying method of CBP (Figure 1). MPGs added by 1% FD (FD 1.0) exhibited the highest CY, indicating superior WHC due to its higher level of added powder and the FD process. MPGs added by 0.5% FD (FD 0.5) and MPGs added by 1% OD (OD 1.0) showed similar improvements to each other, demonstrating a different effect of the drying method (FD, OD). Compared to the control (CTL), MPGs added by 0.5% OD (OD 0.5) improved CY more, but it was less effective than the higher concentrations of OD or the addition of FD. CTL demonstrated the lowest CY, emphasizing the limited WHC of the MPGs in the absence of added proteins. These findings are consistent with a study by Choi and Chin,3 who reported that the addition of plant-based proteins to MPGs can influence CY and WHC.

Regarding GS, FD 1.0 formed the strongest gel network, benefiting from both the FD process and higher concentration of CBP added. OD 1.0 and FD 0.5 showed moderate GS values, with higher concentrations yielding better results. Compared to CTL, OD 0.5 improved GS more, but it remained the weakest among the samples added with CBP. These findings suggest that the addition of FD, particularly at higher concentrations, enhances both the CY and GS of MPGs, likely due to the protein functionality added by CBP. These results align with observations made by Boye et al [11] who reported that freeze-dried legume protein isolates exhibit superior functional properties, including improved water retention and gel formation, compared to those processed using alternative drying methods.

#### Viscosity

**Figure 2:**
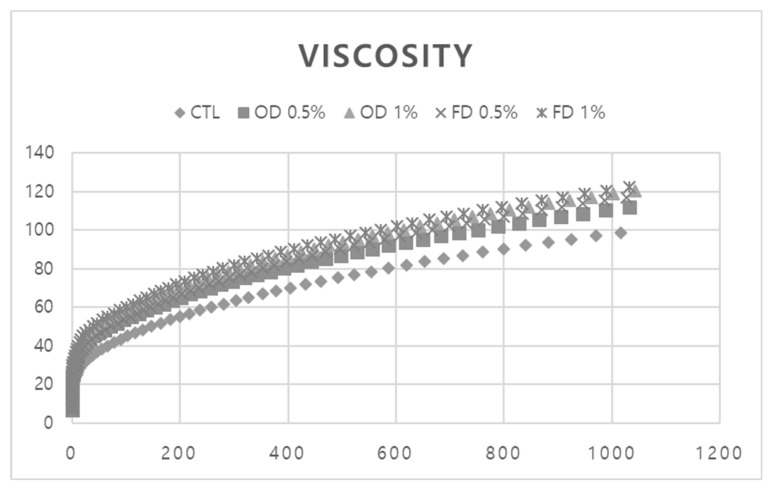
Viscosity of MP gels with different levels of cranberry bean powder processed by different drying methods. Treatment: CTL, control; OD 0.5, MPGs added by 0.5% cranberry bean oven dried powder; OD 1.0, MPGs added by 1.0% cranberry bean oven dried powder; FD 0.5, MPGs added by 0.5% cranberry bean freeze dried powder; FD 1.0, MPGs added by 1.0% cranberry bean freeze dried powder. MP, myofibrillar protein; MPGs, myofibrillar protein gels.

The viscosity of MPGs was influenced by the addition of CBP, with various effects observed depending on the drying method and level (Figure 2). FD 1.0 exhibited the highest viscosity, indicating the formation of a robust protein network and enhanced stability, followed by OD 1.0 and FD 0.5 with moderate viscosity, while OD 0.5 showed a comparatively lower viscosity. CTL demonstrated the lowest viscosity. These findings align with research on other legume flours, such as pinto beans, suggesting that processing methods, such as boiling and sprouting, enhance the water and oil absorption capacities.

The high protein content of cranberry bean flour contributes to its functional properties, as noted by Shevkani et al [13] in their review of pulse proteins. Furthermore, the interaction between plant proteins and polyphenols, as demonstrated with pea protein isolates and cranberry polyphenols by Foegeding et al [14] suggests potential applications for cranberry bean flour in the development of functional food ingredients with improved digestibility and solubility profiles. The superior performance of MPGs added with FD in our study, particularly FD 1.0, underscores the efficiency of FD in preserving and enhancing the functional properties of plant proteins in meat systems. Additionally, the presence of carbohydrates in CBP may contribute to improved gel properties, as Xu and Xu [2] found that the addition of carbohydrates to MPs can result in hydrophobic interactions, electrostatic interactions, or increased hydrogen bonding, potentially enhancing gel formation and stability.

#### Sodium dodecyl sulfate-polyacrylamide gel electrophoresis

**Figure 3:**
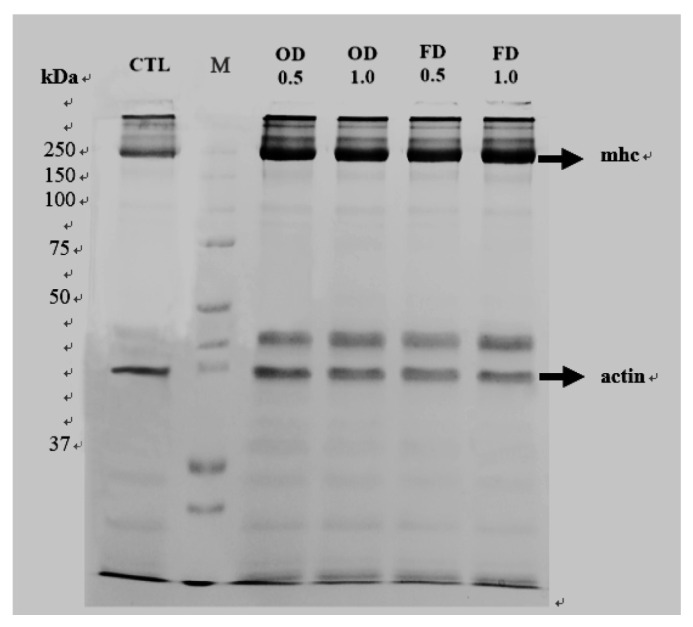
SDS-PAGE profile of MP gels with different levels of cranberry bean powder processed by different drying methods. Treatment: CTL, control; M; standard marker; OD 0.5, MPGs added by 0.5% cranberry bean oven dried powder; OD 1.0, MPGs added by 1.0% cranberry bean oven dried powder; FD 0.5, MPGs added by 0.5% cranberry bean freeze dried powder; FD 1.0, MPGs added by 1.0% cranberry bean freeze dried powder. SDS-PAGE, sodium dodecyl sulfate-polyacrylamide gel electrophoresis; MP, myofibrillar protein; MPGs, myofibrillar protein gels.

Figure 3 presents the SDS-PAGE results of MPGs added with CBP, illustrating the effects of different drying methods. The presence of additional bands, particularly in the lower molecular weight region (below 50 kDa), suggests the incorporation of cranberry bean proteins into the MPG network. While a slight reduction in the intensity of the myosin heavy chain band was observed across samples added with CBP, the samples added with FD generally exhibited sharper, more defined bands compared to those added with OD, indicating better protein integrity when FD, rather than OD, is incorporated. These findings align with research by Kristoffersen et al [15] who used SDS-PAGE to demonstrate that low-molecular-weight proteins can contribute to the gelation and texture of fish muscle, highlighting the importance of protein composition in determining gel properties. The enhanced band clarity in FD and the corresponding MPGs suggest that FD helps preserve the structural integrity of these smaller proteins, potentially contributing to the improved GS and viscosity observed in previous analyses in this study.

#### Microstructure

**Figure 4:**
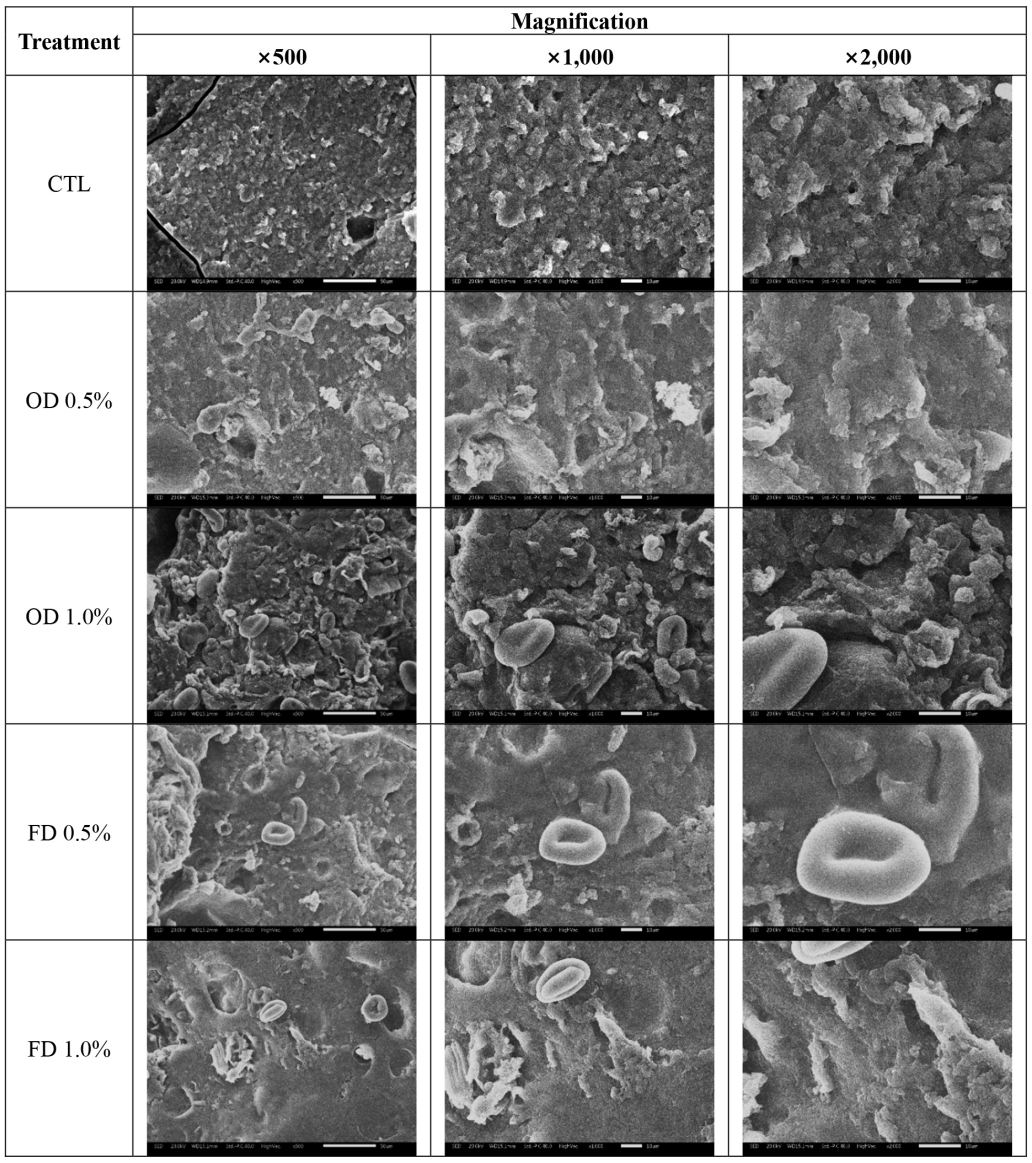
Scanning microscopy of MP gels with different levels of cranberry bean powder processed by different drying methods. Treatment: CTL, control; OD 0.5, MPGs added by 0.5% cranberry bean oven dried powder; OD 1.0, MPGs added by 1.0% cranberry bean oven dried powder; FD 0.5, MPGs added by 0.5% cranberry bean freeze dried powder; FD 1.0, MPGs added by 1.0% cranberry bean freeze dried powder. MP, myofibrillar protein; MPGs, myofibrillar protein gels.

The microstructure analysis of MPGs added with CBP reveals differences among CTL and treated samples (OD 0.5, OD 1.0, FD 0.5, FD 1.0). As shown in Figure 4, bean/egg-like particles, likely starch granules or fiber particles, were observed in the scanning electron microscopy (SEM) images, filling the pores, particularly in the FD samples. This structural arrangement suggests that FD, especially at higher concentrations (FD 1.0), may contribute to improved water retention capacity and enhanced functional properties of the MPGs. Similar bean/egg-like particles were found in a study on the addition of cornstarch to MPGs conducted by Lee and Chin [16].

The addition of starch to MPGs enhances their structural and functional properties. This is attributed to starch’s ability to absorb water and swell, leading to a more compact and homogeneous gel structure. Sun et al [17] observed that the addition of modified starch promoted the heat-induced conformational transition from the α-helix accompanied by the conversion of β-turn to β-sheet, leading to the stretching out of MP molecules. Starch granules act as fillers in the protein network, promoting the conversion of free water to immobilized and bound water, further stabilizing the gel matrix. Hu et al [18] confirmed that the gelling properties of MP from *Lateolabrax japonicus* could be enhanced by starch incorporation, ultrasonic pretreatment, and their synergistic effect. The type and form of starch used can impact MPG properties, with Chen et al [19] reporting that the addition of amylopectin weakened hydrogen bonding and shifted characteristic peaks in the MP composite gels. Interestingly, MPGs added with oven-dried and freeze-dried powders containing starch exhibit pores yet maintain or even improve GS due to the “packing effect” of starch granules, which fill the pores and interact with the protein network, compensating for potential weaknesses in the structure [19]. The presence of these pores, particularly in freeze-dried samples, may contribute to an improved water retention capacity without compromising GS, as the starch granules within the pores provide additional support to the overall gel structure.

### Rheological properties of myofibrillar protein gels added with cranberry bean powder or cranberry bean protein isolate treated with different drying methods

#### Cooking yield and gel strength

**Figure 5:**
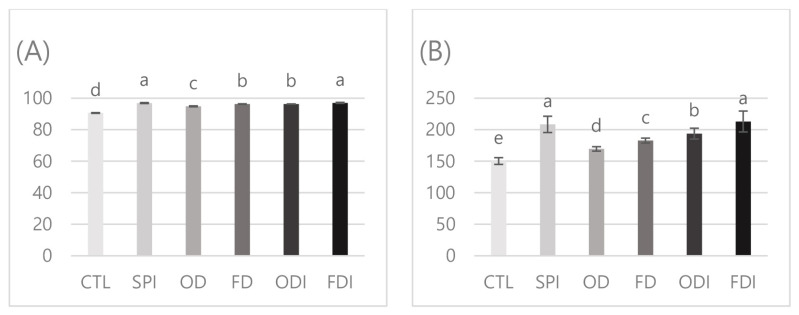
Cooking yield (%) and gel strength (gf) of chicken breast myofibrillar protein gels (MPGs) containing cranberry bean powder or protein isolate processed by different drying methods. (A) Cooking yield, (B) gel strength. Treatment: CTL, control; SPI, MPGs added by 1% of soy protein isolate; OD, MPGs added by 1% of cranberry bean oven dried powder; FD, MPGs added by 1% of cranberry bean freeze-dried powder; ODI, MPGs added by 1% of cranberry bean oven dried protein isolate powder; FDI, MPGs added by 1% of cranberry bean freeze-dried protein isolate powder. ^a–e^ Means with different superscripts in the treatment are different (p<0.05).

The CY and GS results, as shown in Figure 5, demonstrate different variations among the different treatments applied to MPGs. FDI and SPI exhibited the highest CYs and GSs, indicating superior water retention and protein-network formation capabilities. These findings align with research by Hasanpour et al [20] who reported that the addition of soy protein concentrate to surimi gels improved WHC and GS due to enhanced protein-protein interactions and network formation.

In the present study, the protein isolates (FDI and SPI) outperformed other treatments, highlighting their effectiveness in improving both the water retention and gel properties of MPGs. In comparison, ODI and FD treatment showed better performance than OD, as shown in Figure 5, suggesting that CBPI generally yields superior results compared to using CBP. This observation is consistent with a study by Zhang et al [21] who found that protein isolates from various sources could effectively enhance the functional properties of MPGs. The OD treatment exhibited the lowest CY and GS among the treated samples, indicating the limited effect of CBP without protein isolation. CTL consistently showed the lowest values for both CY and GS, reflecting the absence of added proteins and their benefits on water retention and gel formation.

These results emphasize the importance of protein isolation in enhancing the functional properties of MPGs. The superior performance of protein isolates, particularly FDI and SPI, in improving water retention and GS can be attributed to their ability to form stronger protein networks and interact more effectively with MPs. This is supported by Zhang et al [21] who demonstrated that protein isolates can enhance the gelation properties of MPs through increased protein-protein interactions and improved water-binding capacity. Overall, Figure 5 highlights that the addition of CBPI proves to be a more effective strategy for enhancing the water retention and GS of MPGs compared to the addition of CBP. The addition of Citri-Fi 100 (CF) decreased the CY of chicken breast and beef skirt compared with the phosphate (sodium tripolyphosphate, STPP), and this result was highly associated with the fat- and WHC [22].

#### Viscosity

**Figure 6:**
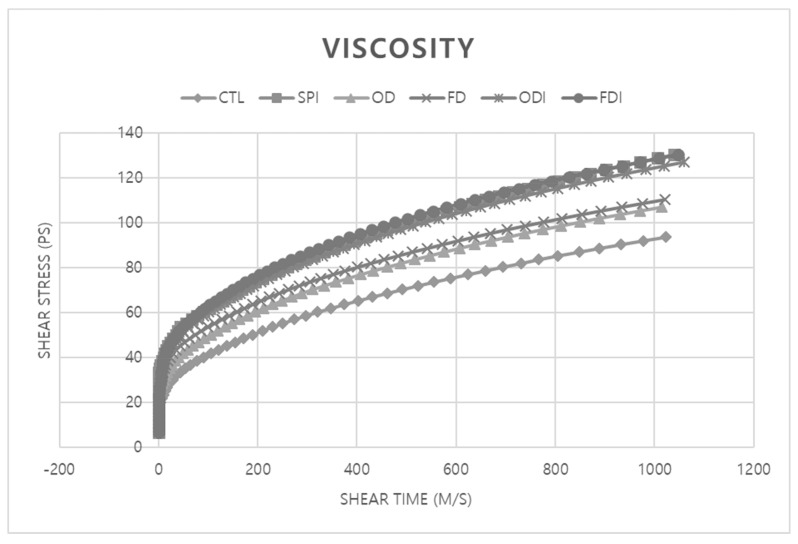
Viscosity of MP gels with cranberry bean powder and protein isolate processed by different drying methods. Treatment: CTL, control; SPI, MPGs added by 1% of soy protein isolate; OD, MPGs added by 1% of cranberry bean oven dried powder; FD, MPGs added by 1% of cranberry bean freeze-dried powder; ODI, MPGs added by 1% of cranberry bean oven dried protein isolate powder; FDI, MPGs added by 1% of cranberry bean freeze-dried protein isolate powder. MP, myofibrillar protein; MPGs, myofibrillar protein gels.

The viscosity results, as shown in Figure 6, demonstrate clear differences among the various treatments applied to MPGs. FDI and SPI exhibited the highest viscosity values, indicating superior protein-network formation and stability. This aligns with their enhanced GS and water retention properties, as higher viscosity often correlates with improved gel characteristics. ODI showed slightly lower viscosity than FDI and SPI but still demonstrated enhancement compared to other treatments. FD and OD treatments displayed moderate viscosity, with FD outperforming OD, suggesting that FD may better preserve protein functionality. CTL consistently showed the lowest viscosity, reflecting poor protein interaction and water retention due to the absence of added proteins. These findings are consistent with research by Niu et al [23] who reported that the addition of modified SPI to MPGs increased compression force and decreased water loss while also enhancing hydrogen bonding and disulfide linkages. Similarly, Jang et al [24] found that the addition of red bean protein isolate to MPGs increased CY and improved rheological properties, especially when combined with microbial transglutaminase. These studies support the observed trend in Figure 6, where protein isolates enhance the rheological properties of MPGs compared to CBP and CTL.

#### Sodium dodecyl sulfate-polyacrylamide gel electrophoresis

**Figure 7:**
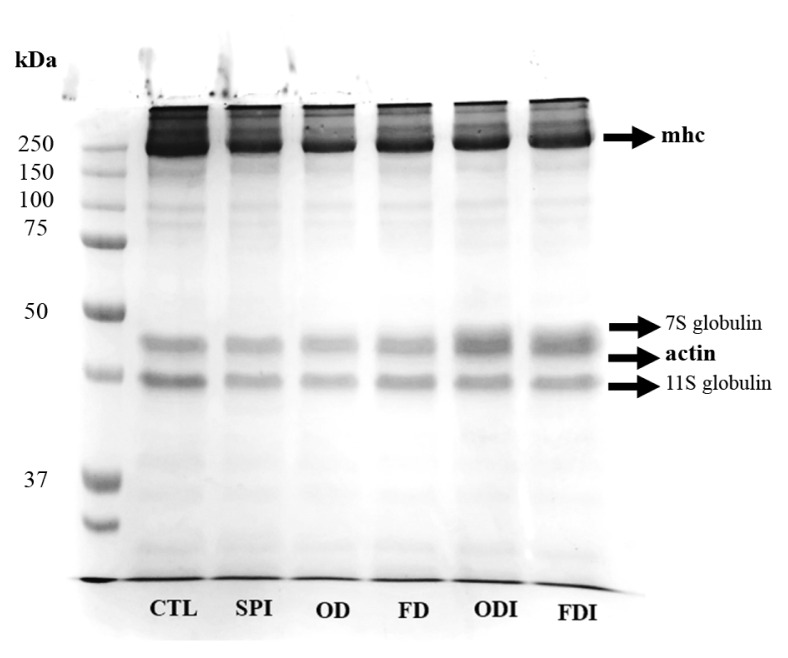
SDS-PAGE profile of MP gels with cranberry bean powder and protein isolate processed by different drying methods. Treatment: CTL, control; SPI, MPGs added by 1% of soy protein isolate; OD, MPGs added by 1% of cranberry bean oven dried powder; FD, MPGs added by 1% of cranberry bean freeze-dried powder; ODI, MPGs added by 1% of cranberry bean oven dried protein isolate powder; FDI, MPGs added by 1% of cranberry bean freeze-dried protein isolate powder. SDS-PAGE, sodium dodecyl sulfate-polyacrylamide gel electrophoresis; MP, myofibrillar protein; MPGs, myofibrillar protein gels.

The SDS-PAGE results, as shown in Figure 7, reveal distinct patterns of protein interaction and solubility across different treatments applied to MPGs. While the myosin heavy chain (~200 kDa) band remained consistent across all samples, its intensity slightly decreased in the OD and FD treatments, suggesting less efficient protein interaction compared to the ODI, FDI, and SPI samples. The actin (~42 kDa) band remained stable, indicating that the core myofibrillar structure was unaffected by the addition of CBP. Notably, new bands in the 40–60 kDa range were observed in the ODI and FDI samples, corresponding to plant proteins, with FDI exhibiting the sharpest and most defined bands.

These new bands could potentially represent 7S or 11S globulins, which are known to enhance GS through interactions with myosin. This observation is consistent with the findings of Bühler et al [25] who identified bands corresponding to vicilin (7S globulin) at 46–55 kDa and α-legumin (11S globulin) at 38–40 kDa in dry-heated and non-dry-heated faba bean protein concentrates. These globulins have been shown to improve gel formation by interacting with myosin, suggesting that the inclusion of ODI or FDI may alter protein structure and enhance the functional properties of MPGs. In chicken meat, the chemical modification of methionine oxidation highly plays a critical role in the mechanisms associated with growth-related myopathies [26].

#### Microstructure

**Figure 8:**
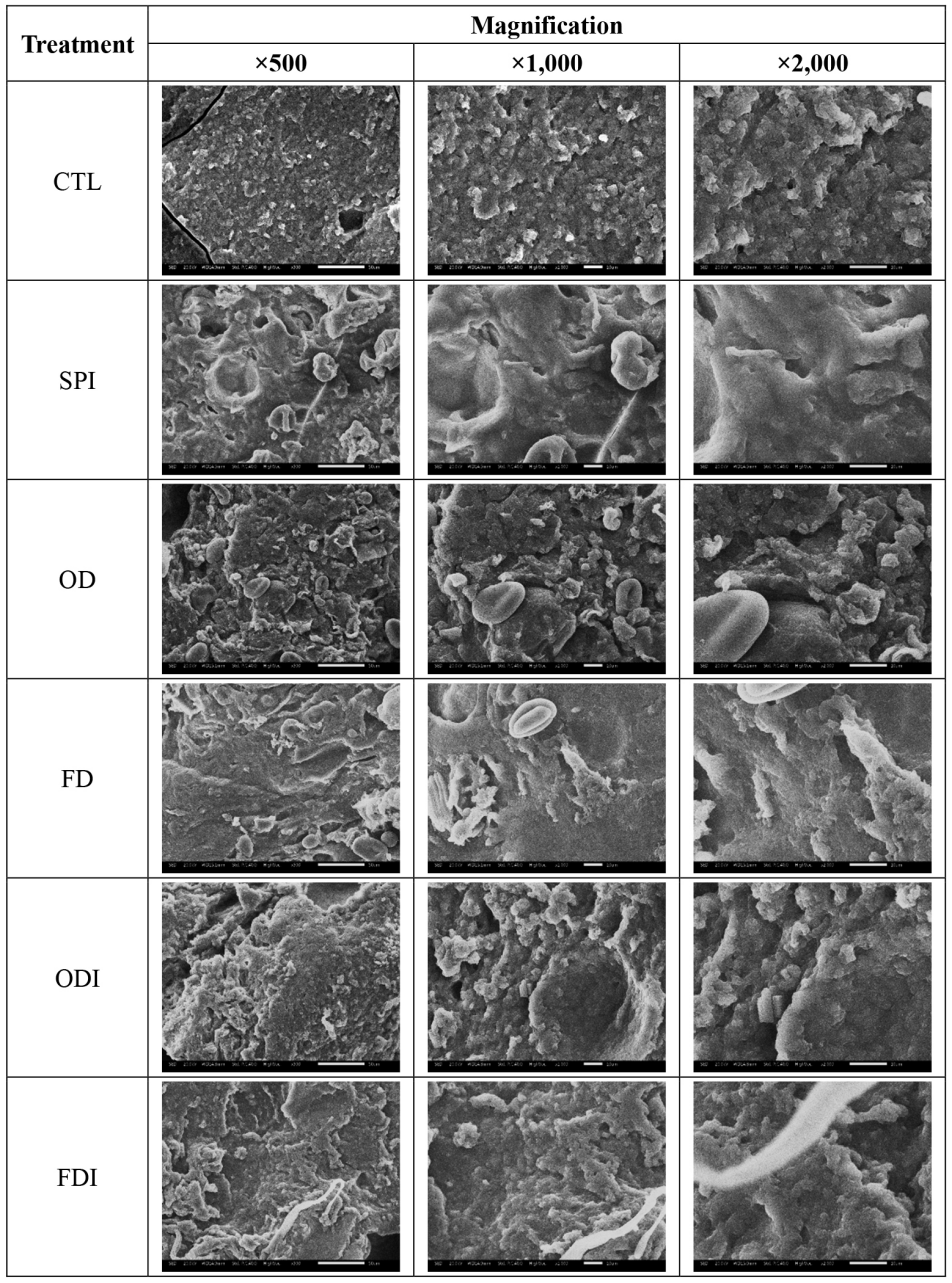
Scanning microscopy of MP gels with cranberry bean powder and protein isolate processed by different drying methods. Treatment: CTL, control; SPI, MPGs added by 1% of soy protein isolate; OD, MPGs added by 1% of cranberry bean oven dried powder; FD, MPGs added by 1% of cranberry bean freeze-dried powder; ODI, MPGs added by 1% of cranberry bean oven dried protein isolate powder; FDI, MPGs added by 1% of cranberry bean freeze-dried protein isolate powder. MP, myofibrillar protein; MPGs, myofibrillar protein gels.

The SEM analysis, as shown in Figure 8, revealed distinct microstructural differences among the various treatments. The FD and OD samples exhibited bean/egg-like structures, likely representing starch granules or fiber particles, which correlate with the high carbohydrate content of these powders. In contrast, the ODI and FDI samples displayed more compact structures with fewer and smaller pores, similar to the SPI sample. These results suggest that protein isolates, particularly ODI and FDI, form more stable networks with reduced pore formation compared to OD and FD, resulting in improved texture and GS. These findings align with research by Lorenzen and Schrader [27] who reported that whey protein isolates formed stronger and more elastic gels than whey protein concentrates, attributing this to the higher protein content and lower levels of non-protein components. Similarly, Tang et al [28] found that whey protein isolates gels with higher WHC had smaller pores and a more uniform structure, whereas gels with lower WHC exhibited larger pores and reduced GS. Overall, microstructural analysis suggests that the protein isolates (ODI and FDI) are comparable to SPI in their ability to reduce pore size and enhance the gel matrix. In comparison, OD and FD exhibit greater porosity, which may contribute to higher water retention but weaker network.

## CONCLUSION

This study revealed that FDI demonstrated the best performance among all treatments, showing comparable GS, CY, and viscosity to SPI. FDI exhibited enhanced protein-protein interactions, as supported by SDS-PAGE and SEM analyses, where it formed well-structured, elongated protein networks similar to SPI. In contrast, OD and FD despite containing higher levels of carbohydrates and starch, showed varied interactions with MP. OD displayed reduced GS and less stable gel networks, likely due to the interference of starch in protein matrix formation. Conversely, FD performed better than OD, with improved GS and viscosity, but it was still not as effective as FDI. The differences between drying methods highlight the importance of preserving protein structure, as FD more effectively maintains the functional properties of cranberry bean protein, making it a more promising ingredient for enhancing MP gelation compared to OD.

## Figures and Tables

**Table 1:** Formulation of chicken breast MPGs at various levels of cranberry bean powder with different drying methods

Ingredient (%)	Treatment^1)^				

	CTL	OD 0.5	OD 1.0	FD 0.5	FD 1.0
MP mixture	80	80	80	80	80
Buffer solution	20	19.5	19	19.5	19
Cranberry bean powder	0	0.5	1.0	0.5	1.0
Total	100	100	100	100	100

1) Treatment: CTL, control; OD 0.5, MPGs added by 0.5% cranberry bean oven dried powder; OD 1.0, MPGs added by 1.0% cranberry bean oven dried powder; FD 0.5, MPGs added by 0.5% cranberry bean freeze dried powder; FD 1.0, MPGs added by 1.0% cranberry bean freeze dried powder.

MPGs, myofibrillar protein gels; MP, myofibrillar protein.

**Table 2:** Formulation of chicken breast MPGs added with cranberry bean powder and isolates with different drying methods

Ingredient (%)	Treatment^1)^					

	CTL	SPI	OD	FD	ODI	FDI
MP mixture	80	80	80	80	80	80
Buffer solution	20	19	19	19	19	19
Sample powder	0	1.0	1.0	1.0	1.0	1.0
Total	100	100	100	100	100	100

1) Treatment: CTL, control; SPI, MPGs added by 1% of Soy protein isolate; OD, MPGs added by 1% of cranberry bean oven dried powder; FD, MPGs added by 1% of cranberry bean freeze-dried powder; ODI, MPGs added by 1% of cranberry bean oven dried protein isolate powder; FDI, MPGs added by 1% of cranberry bean freeze-dried protein isolate powder.

MPGs, myofibrillar protein gels; MP, myofibrillar protein.

**Table 3:** Chemical composition cranberry bean powder and isolates with different drying methods

Treatment^1)^	Chemical composition (%)			

	Moisture	Lipid	Protein	Ash
OD	10.34±1.15^a^	1.9±0.50^b^	21.73±0.87^d^	3.72±0.04^b^
FD	6.36±0.42^b^	2.53±0.16^a^	23.99±0.41^c^	3.91±0.14^c^
ODI	2.13±0.47^c^	0.01±0.01^c^	86.45±0.40^b^	5.98±0.90^a^
FDI	2.88±0.46^c^	0.01±0.00^c^	91.97±0.38^a^	4.73±0.7^a^

1) Treatment: OD, MPGs added by 1% of cranberry bean oven dried powder; FD, MPGs added by 1% of cranberry bean freeze-dried powder; ODI, MPGs added by 1% of cranberry bean oven dried protein isolate powder; FDI, MPGs added by 1% of cranberry bean freeze-dried protein isolate powder.

a–d Means with different superscripts in the treatment are different (p<0.05).

MPGs, myofibrillar protein gels.

## Data Availability

Upon reasonable request, the datasets of this study can be available from the corresponding author.
